# Survival improvements in esophageal and gastric cancers in the Nordic countries favor younger patients

**DOI:** 10.1002/cam4.7365

**Published:** 2024-08-03

**Authors:** Kari Hemminki, Frantisek Zitricky, Asta Försti, Otto Hemminki, Vaclav Liska, Akseli Hemminki

**Affiliations:** ^1^ Biomedical Center, Faculty of Medicine in Pilsen Charles University Pilsen Czech Republic; ^2^ Division of Cancer Epidemiology German Cancer Research Center (DKFZ) Heidelberg Germany; ^3^ Hopp Children's Cancer Center (KiTZ) Heidelberg Germany; ^4^ Division of Pediatric Neurooncology German Cancer Research Center (DKFZ), German Cancer Consortium (DKTK) Heidelberg Germany; ^5^ Department of Urology Helsinki University Hospital and University of Helsinki Helsinki Finland; ^6^ Cancer Gene Therapy Group, Translational Immunology Research Program University of Helsinki Helsinki Finland; ^7^ Department of Surgery, University Hospital, Faculty of Medicine in Pilsen Charles University Pilsen Czech Republic; ^8^ Comprehensive Cancer Center Helsinki University Hospital Helsinki Finland

**Keywords:** mortality, relative survival, risk factors, stomach cancer, treatment

## Abstract

Esophageal cancer (EC) and gastric cancer (GC) are fatal cancers with a relatively late age of onset. Age is a negative risk factor for survival in many cancers and our aim was to analyze age‐specific survival in EC and GC using the recently updated NORDCAN database. NORDCAN data originate from the Danish, Finnish, Norwegian, and Swedish nationwide cancer registries covering years 1972 through 2021 inviting for comparison of 50‐year survival trends between the countries. Relative 1‐ and 5‐year survival and 5/1‐year conditional survival (i.e., survival in those who were alive in Year 1 to survive additional 4 years) were analyzed. Survival in EC showed large gains for patients below age 80 years, 5‐year survival in Norwegian men reaching 30% and in women over 30% but for 80–89 year old survival remained at 10%. In contrast, hardly any gain was seen among the 80–89 year patients for 1‐year survival and small gains in 5 year and 5/1‐year survival. Survival gaps between age‐groups increased over time. For GC there was also a clear age‐related negative survival gradient but the survival gaps between the age groups did not widen over time; Norwegian male and female 5‐year survival for 80–89 year old was about 20%. The age‐specific survival difference in GC arose in Year 1 and did not essentially increase in 5‐year survival. While there were differences in survival improvements between the countries, poor survival of the 80–89 year old patients was shared by all of them. To conclude, survival has improved steadily in younger GC and EC patients in most Nordic countries. While the 80–89 year old population accounts for nearly a quarter of all patients and their poor survival depressed overall survival, which can therefore be increased further by improving diagnostics, treatment and care of elderly EC and GC patients.

## Graphical abstract

The data provide up‐to‐data relative survival data from esophageal and gastric cancers in age‐groups. Survival gaps are increasing between young and old patients.

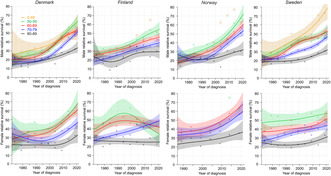

## 1 INTRODUCTION

Survival in gastric cancer (GC) and esophageal cancer (EC) has improved but EC remains (with pancreatic and pleural cancers) the most fatal solid cancer, and survival in GC is only marginally better.^1^, ^2^, ^3^ The oldest national survival data are available from the Nordic countries where 5‐year relative survival in 1964–1968 varied between 7% and 14% for GC and 0% and 6% for EC.^4^ Historically, squamous cell histology was dominant for both of these cancers but in EC adenocarcinoma has become the main histological type in Europe and North America, most likely because of obesity related gastroesophageal reflux disease.^5^ GC and EC are often treated in the same clinics because similar methods are used for diagnostics and treatment.^6^, ^7^

A large proportion of EC is diagnosed in the lower 1/3 of the esophagus or at the gastroesophageal junction while GC is commonly found in the corpus or the antrum.^8^ Both of the cancers are typically diagnosed at an advanced stage (IV) and T3 is the most common T class in Sweden.^8^ For early stage disease endoscopic techniques have increasingly been applied while for advanced disease open surgery has remained the standard treatment, supplemented more recently with neoadjuvant chemotherapy or chemoradiation.^7^, ^8^ In Sweden resection rates for GC and EC were close to 40% and 30%, respectively, in 2007, and slightly declined subsequently; neoadjuvant therapy for resected patients markedly increased with time, reaching 80% for EC and 50% for GC patients in 2018 (https://statistik.incanet.se/Esofagus‐Ventrikel/). Chemotherapy remains the backbone of treatment of metastatic GC and EC, but immunotherapy and anti‐Her2 therapies, and operation or ablation of liver metastases have recently been incorporated into treatment algorithms, but these could influence only for the last years.^9^, ^10^ Treatment has been age‐dependent and according to the Swedish national quality register curative treatment in 2007–18 for EC was offered to 50%–60% of patients below age 70 years, to 40%–50% for those aged 70–79 but to only 15% of older patients (https://statistik.incanet.se/Esofagus‐Ventrikel/). For GC the proportions were similar for the younger patients but for 70–79 year‐old the percentage was 50% and for the older it was 25%. In the last decades treatment of these cancers has been centralized to expert clinics, care pathways have been facilitated and most patients are seen by multidisciplinary teams.^7^, ^8^

Cancer survival depends on many factors of which stage and age at diagnosis are the main determinants for most tumor types.^11^, ^12^ While the stage is directly related to spreading, the influence of age may be complex even in stage‐specific analysis, with possible influence of treatment, socio‐economic factors, comorbidities and frailty, assessed by performance status.^13^, ^14^ Furthermore, age is often adjusted in the analysis erasing its contribution. The proportion of cancer patients diagnosed at age over 80 years is projected to increase.^15^ Comparison of age differences in survival over a long period of time and between countries may help to understand causes for the differences and suggest actions to correct them.

Data available in the Nordic countries with a long‐term tradition of high‐quality cancer registration covering the whole population are particularly suitable for such analysis. An additional condition for population‐level survival analysis is citizens' universal access to health care which is fulfilled in the Nordic countries. However even though the Nordic counties have organized their health care largely in a similar way, the available resources have varied in time (Table S1). In 1970, the Finnish and Norwegian gross national product (GNP) per person was about a half of that of Denmark and Sweden which even used a larger share for health care. In 2010 the share of health care spending was highest in Denmark but considering the higher GNP in Norway the absolute spending was about equal between these countries, ahead of Finland, and further ahead of Sweden. The Finnish GNP remained below others and the share for health case was also among the lowest.

The Nordic cancer registries transferred recently their long‐term incidence, mortality and survival data to the International Agency for Research on Cancer (IARC) allowing public access (https://nordcan.iarc.fr/en). We use this NORCAN dataset to model age‐ and sex‐specific 1‐ and 5‐year relative survival and conditional 5/1‐year survival (i.e., survival for those who were alive in year 1 to survive the next 4 years) for GC and EC over a 50‐year period in Denmark (DK), Finland (FI), Norway (NO) and Sweden (SE). Our aim is to interpret the country‐specific results in terms of critical survival periods (i.e., times when survival changes in a short period), factors contributing to age‐ and sex‐ specific survival and suggestions to curb age disadvantages. For comparison we show survival data for these cancer from USA.

## 2 MATERIALS AND METHODS

The NORDCAN database 2.0 was accessed at the IARC website (https://nordcan.iarc.fr/en) in fall of 2023.^16^, ^17^, ^18^ The available data are grouped and not based on individual patients. Age‐groups were as defined in NORDCAN. We extracted data on case numbers and 1‐and 5‐year relative age‐specific survival (i.e., relative survival in patients who were diagnosed at the defined age brackets) from 1972 until the end of 2021. Additionally conditional 5/1‐year survival was estimated for patients who had survived the first year for surviving additional 4 years. Survival method was the “hybrid” analysis to ensure the most recent survival information; cohort method was used to all but the last 5‐year period for which the period method was used.^19^ Age‐specific relative survival was estimated using the Pohar Perme estimator.^20^ The survival estimates for all age groups combined were age‐standardized using ICSS weights by Brenner's method.^21^ National general population life‐tables stratified by sex, year and age were used in the calculation of expected survival. Exclusions included patients with only death certificate data and those 90 years or older. Inclusion criteria for age‐specific survival specified that a minimum of 30 patients were alive at start, three patients alive criterion in each weight group for age standardized.

Statistical analysis was performed using R statistical software (https://www.r‐project.org) in the R studio environment (https://posit.co/). The trends in relative survival were modeled using Bayesian generalized additive models, as described previously,^22^ using an adopted version of the code (https://github.com/filip‐tichanek/nord_intestine). The modeling was performed on cumulative hazard scale, permitting asymmetric confidence intervals (CIs) of individual NORDCAN estimates. We fitted separate models for each metric and each country, for EC and GC. The models included the main effect of the group (combination of age‐specific groups and sex) and non‐linear effect of time, modeled with thin plate regression splines (mgcv package, *k* = 5). The female 60–69 years group was modeled as a reference level (intercept) with effects of the other groups having Gaussian prior (mean = 0, sigma = 1 for cumulative hazard related to 1‐year and 5/1‐year relative survival; 5‐year relative survival: sigma = 3 (EC) and sigma = 2 (GC)). For the purpose of modeling of temporal trends, the relative survival estimates for individual 5‐year periods were assigned timepoint in the middle of the respective period. The modeled trends were visualized after back‐transforming from cumulative excess hazard scale to relative survival scale.

Case numbers for some EC subgroups are small, warranting caution. The uncertainty in estimates due to small case numbers is addressed in our model of survival trends, which considers both the survival estimates and their associated standard errors as the model input. Overfitting is reduced by including penalty terms for curve wiggliness, determined a by cross‐validation procedure.

Survival data from USA was accessed at the US Surveillance, Epidemiology and End Results (SEER) web site for years 2015–2019 for Whites and Hispanics (https://seer.cancer.gov/statistics‐network/explorer/application.html?site=1&data_type=1&graph_type=2&compareBy=sex&chk_sex_3=3&chk_sex_2=2&rate_type=2&race=1&age_range=1&hdn_stage=101&advopt_precision=1&advopt_show_ci=on&hdn_view=0&advopt_display=2#graphArea).

Differences were called significant when the 95% CIs were nonoverlapping.

## 3 RESULTS

Case numbers for all and 80–89‐year‐old patients and median ages of onset for EC and GC in two periods (1972–1976 and 2017–2021) are shown in Table 1. For EC in men, overall case numbers increased between twofold (SE) and fourfold (DK) and the proportion of old patients increased slightly more and thus the median age of onset also increased from 67–69 years to 70–71 years. For EC in women, the increase in case numbers was less (none in FI) and the median age at onset decreased from 73–75 years to 72–74 years. The oldest (80–89 years) EC patients accounted for 16.2% of all male and 24.9% of all female patients; the proportion did not change in 50 years. Case numbers for male GC were initially far higher than those for EC but in the last period the difference was small because of the opposite incidence trends (decline in GC, increase in EC). The median age of onset for GC in 2017–2021 was 71–74 years. For female GC the decrease in case numbers was also large and the median age of onset was 72–74 years. In 2017–2021, the 80–89 year old GC patients accounted for 23.1% of all male and 28.8% of all female patients; the proportions increased over the 50 year time span of the study.

**TABLE 1:** Numbers of all esophageal and gastric cancer patients, 80–89 year old and median ages of onset in 1972–1976 and 2016–2021.

Esophageal cancer								
Country	Men 1972–1976	80–89 year	%, old	Age	Men 2017–21	80–89 year	%, old	Age
Denmark	513	72	14	69	2062	327	15.9	71
Finland	487	65	13.3	68	1328	177	13.3	70
Norway	404	70	17.3	67	1237	206	16.7	71
Sweden	996	149	15	69	1868	354	19	71

Country	Women 1972–1976	80–89 year	%, old	Age	Women 2017–21	80–89 year	%, old	Age
Denmark	288	96	33.3	75	735	134	18.2	72
Finland	618	150	24.3	73	511	163	31.9	74
Norway	154	32	20.8	75	430	116	23.3	73
Sweden	449	132	29.4	75	725	190	26.2	73

Gastric cancer								
Country	Men 1972–1976	80–89 year	%, old	Age	Men 2017–21	80–89 year	%, old	Age
Denmark	3446	761	22.1	73	2538	503	19.8	71
Finland	3647	378	10.4	68	1895	435	23.1	71
Norway	3243	565	17.4	70	1347	352	26.1	72
Sweden	6393	1270	19.9	70	2631	618	23.5	73

Country	Women 1972–1976	80–89 year	%, old	Age	Women 2017–21	80–89 year	%, old	Age
Denmark	2347	716	30.5	76	1138	269	23.6	72
Finland	2902	588	20.2	72	1311	401	30.6	73
Norway	2089	530	25.4	74	873	275	31.5	74
Sweden	3989	1155	29	74	1569	463	29.5	73

### 3.1 Survival in EC

**FIGURE 1:**
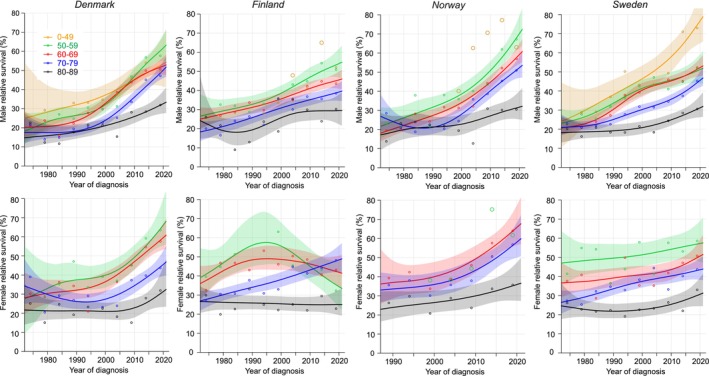
Relative 1‐year survival with 95% CIs in esophageal cancer among Nordic men and women between 1972 and 2021. If case numbers were too few for complete curves, the available individual data points were shown with large circles (orange: 0–49 years, green: 50–59 years).

Age‐specific 1‐year survival for EC is described in Figure 1 for Nordic men and women. Note that for the age‐group below 50 years case numbers were few and full curves could be plotted for DK and SE men only; for FI and NO men individual data points are shown; for women this age‐group is not included. 95% CIs are shown for all curves enabling visual assessment of the significance.

In Figure 1 male survival in DK and NO started to increase linearly since about 1995 and in NO reached 70% in the 50–59‐year‐old. Men in FI and SE at this age reached a survival of over and under 50%, respectively. In SE, men below age 50 years reached an almost 80% survival. For the oldest men (80–89 years) survival reached the 30% mark. Female survival in DK resembled male survival but in NO and SE it was higher compared to men; NO survival for the oldest women reached 35%. Female survival for the youngest FI patients (50–55 years) declined after year 1990. According to the NORDCAN data on EC combining all age‐groups, overall 1‐year relative survival in 2017–2021 was best in NO men (53.7%) and women (57.3%), significantly better than in FI (42.4% and 44.9%, respectively).

**FIGURE 2:**
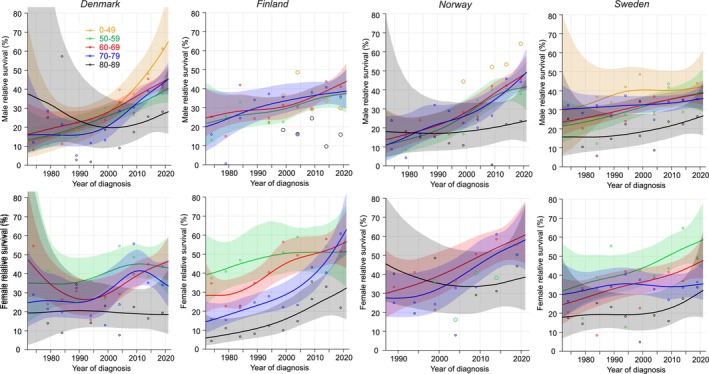
Relative 5/1‐year conditional survival with 95% CIs in esophageal cancer among Nordic men and women between 1972 and 2021. If case numbers were too few for complete curves, the available individual data points were shown with large circles (orange: 0–49 years, green: 50–59 years, black: 80–89 years).

Survival in EC in years 2 to 5 (5/1‐year conditional survival) is shown in Figure 2. Survival increased in DK men and women first after 1990 and then increased steeply for men; for women the increase stalled in 2010. Survival increased in FI and NO men and women except for NO 80‐89‐year‐old patients.

**FIGURE 3:**
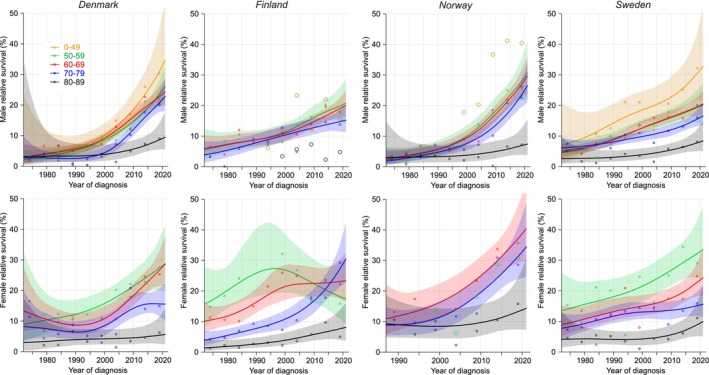
Relative 5‐year survival with 95% CIs in esophageal cancer among Nordic men and women between 1972 and 2021. If case numbers were too few for complete curves, the available individual data points were shown with large circles (orange: 0–49 years, green: 50–59 years, black: 80–89 years).

Age‐specific 5‐year relative survival (Figure 3) is the synthesis of 1‐ and 5/1‐year survival. For DK and NO men survival was below 10% until about year 2000 and then increased steeply in all age‐groups; young patients (below 59 years) reached 30% survival. The 80–89‐year‐old never reached 10% survival, in agreement with FI and SE. For FI and SE men the development was more linear and steepest for young patients. Female survival increased in all age groups but most among young patients resulting in widening of age differences. For 50–60‐year‐old FI women survival culminated before year 2000 because 1‐year survival culminated at that time (Figure 1). According to the NORDCAN data on all ages, 5‐year survival in 2017–2021 was best in NO men (23.9%) and women (30.8%), significantly better than in FI men (14.4%) and DK women (19.59%).

Overall 5‐year survival in EC according to US SEER database (2015) was 22.0% for men (adenocarcinoma 23.5%, squamous cell carcinoma 18.9%) and 23.0% for women (adenocarcinoma 21.5%, squamous cell carcinoma 25.5%). Adenocarcinoma accounted for 80% of male and 54% of female EC. The available age‐groups were <50, 50–64 and 65+ years; for men, in the age‐groups, EC survival was 25.8%, 22.8%, and 20.9%; for women it was 27.8%, 28.6%, and 19.9%. None of the age‐groups or sex differences were significant.

### 3.2 Survival in GC

**FIGURE 4:**
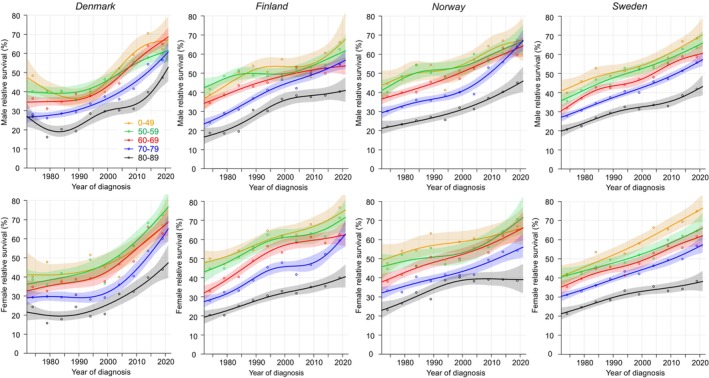
Relative 1‐year survival with 95% CIs in gastric cancer among Nordic men and women between 1972 and 2021.

Age‐specific 1‐year survival in GC is shown in Figure 4. The curves for DK men and women were flat or even decreased till 1995 and then steeply increased reaching almost 70% for the younger and over 50% for the oldest men. For FI men the improvement stalled in the 1990s and resumed later at a slower tempo; the oldest men reached 40% survival. In NO and SE survival increased continuously, with younger men approaching the 70% level and the oldest crossing the 40% level. The shapes of the female survival curves resembled the male ones and were somewhat better than the male ones. According to the NORDCAN data on GC combining all age‐groups, overall 1‐year survival in 2017–2021 was best in NO men (60.7%) and DK and FI women (shared 61.4%).

**FIGURE 5:**
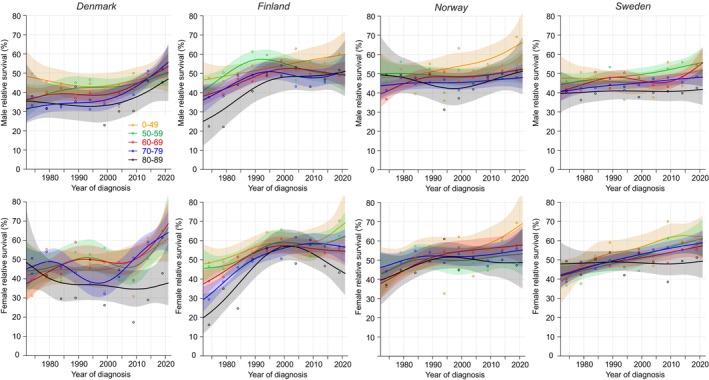
Relative 5/1‐year conditional survival with 95% CIs in gastric cancer among Nordic men and women between 1972 and 2021.

Age‐specific 5/1‐year conditional survival curves for GC in men and women were tightly bundled, in many periods within 10% units from each other (Figure 5). Survival in NO and SE men and women was at 40–50%. In DK, survival curves were flat until they shot upwards after year 2000 (except for old women). In FI the curves culminated for men at around year 1995 and for women at year 2000.

**FIGURE 6:**
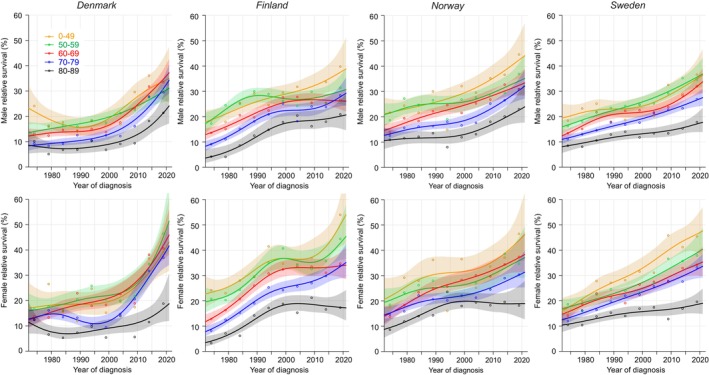
Relative 5‐year survival with 95% CIs in gastric cancer among Nordic men and women between 1972 and 2021.

Because 5‐year survival is the synthesis of 1‐and 5/1‐year survival, the shapes of the male and female survival curves in Figure 6 were like the curves in Figure 4 but drawn at 20%–40% unit lower y‐axis scale. In FI survival increased initially well but culminated temporarily before year 2000, due to the culmination in 5/1‐year survival. Among all GC patients, survival for the 80–89‐year‐old improved through the 50 years but remained at 20% or below. According to the NORDCAN data on GC, overall 5‐year survival in 2017–21 was best in NO men (32.3%) and DK women (37.1%).

Overall 5‐year survival in GC according to US SEER database (2015) was 29.9% for men and 39.2% for women (the difference was significant). In age‐groups <50, 50–64 and 65+ years male GC survival was 34.7, 31.6 and 27.8%; for women it was 39.6%, 46.6%, and 34.9% (the difference between last two figures was significant).

### 3.3 Sex‐specific 5‐year survival

In period 2017–21 5‐year survival in EC was significantly better for FI women compared to men; male survival 14.4% (12.2%–17.1%) versus female 24.8% (19.8%–31.2%). For GC survival in DK and FI showed female advantage; DK male 29.5% (27.3%–31.9%) versus female 37.1% (33.9%–40.6%) and FI male 28.7% (26.2%–31.4%) versus female 35.9 (32.9%–39.1%). Even in NO and SE female survival for both cancer exceeded male survival but the differences were not significant.

Age‐ and sex‐specific 5‐year survival in 2017‐21 was analyzed in Table S2. The only significant difference for EC was female advantage (more than twofold difference) in FI for 70–79‐year old. In all countries female survival exceeded (non‐significantly) male survival particularly in age‐groups below 70 years. For GC female survival in DK 50–59‐year‐old was significantly better that male survival. Similar to EC, female GC survival advantage was evident in younger age‐groups.

## 4 DISCUSSION

The aims of the present study were to analyze age‐specific survival in EC and GC and to estimate the critical periods for patient outcomes. We were able to achieve this task by using updated survival data from the NORDCAN database until year 2021. The overall development in survival over the 50 years has generally been positive: for EC 1‐year survival has approximately doubled to about 50% and 5‐year survival has increased from <10 to 20%, with country‐specific differences. A notable exception was FI, where 1‐year survival and, to a lesser extent 5‐year survival, showed decreasing tendency in the 50–69‐year‐old women since the early 1990s. This is unexplained finding because survival increased among 70‐79‐year‐old women and actually the survival curves of these age‐groups crossed. However, resulting trends were associated with a large posterior uncertainty in the young patient group related to small case numbers. Survival increased for FI men, although less than for the other Nordic men. For GC, historical survival has been somewhat better than that for EC and the small edge was maintained; 1‐year survival has reached 60% and 5‐year survival has more than doubled to over 30% being somewhat better for women compared to men.

Age‐specific improvement in EC showed a similar tendency for 1‐ and 5‐year survival, with large gains for younger patients but hardly any gain among the 80–89‐year old for 1‐year survival and a small gain in 5‐year survival. For GC there was also a clear age‐related negative gradient but the survival gaps between the age groups did not widen as they did for EC because survival in GC from Year 2 to 5 was quite homogeneous between the age‐groups. Thus the age‐group specific 5‐year survival was almost a replica of the 1‐year survival with survival levels halved. The paradox in the between‐country comparison was that even though the overall 5‐year survival in EC and GC was best in NO men, NO old males fared no better than their Nordic counterparts. Similarly, DK overall excellence in 5‐year survival in female GC did not translate to any advantage for old DK women.

Survival in EC in 1972–1976 was low and age‐group differences were small. Such differences arose with time, largely in 1‐year and to a lesser extent in 5/1‐year survival, accounting for the marked age disparity by 2017–2021. For GC, the age‐specific survival gradient was almost entirely due to 1‐year survival; it was already present in 1972–1976 and was maintained through the 50 years. The results would be consistent with the notion that the marked 1‐year survival increase was achieved with novel diagnostic methods and treatment which benefitted younger patients and helped them survive Year 1 but many succumbed before reaching 5 year.^3^ Poor survival among old patients is ascribed to less active diagnosis and thus higher stage impeding treatment, which is also limited by comorbidities.^13^, ^23^, ^24^ According to the Swedish quality register, curative treatment in 2007–2018 for GC and EC was offered to 40%–60% of patients below age 80, but for older patients only to 25% of GC and 15% of EC patients (https://statistik.incanet.se/Esofagus‐Ventrikel/). This is in line with better 5‐year survival figures for 80–89‐year old GC patients compared to EC patients.

In order to understand survival changes over time we need to observe that the survival improvements for GC stated already in the beginning of the study in the 1970s while for EC survival started to improve around year 2000 in DK and NO, and somewhat earlier in FI and SE. For GC in the early period of the present study <1/3 of the patients were treated with curative intent involving surgical resection of the primary tumor and the regional lymph nodes.^25^ Endoscopic resections were developed for early GC. For the majority of patients, chemotherapy and/or radiotherapy was offered as adjuvant therapy with surgery or in the palliative setting.^25^ Improvements in GC have been ascribed to more centralized treatment with lower early mortality and more effective adjuvant treatment, used in increasing proportion of patients.^4^, ^8^ The current treatment guidelines for local GC recommend preoperative and postoperative chemotherapy; for metastatic GC combinations of chemotherapy and anti‐PD1 immunotherapy are recommended.^10^ Surgical treatment for EC has been demanding because it has been among of largest surgical procedures carried out.^26^ Thus mortality has been high calling for experienced specialized surgery. Most likely the late increase in EC survival is explained by the demands for high surgical quality which were achieved by highly specialized treatment centers from year 2000 onward.^8^ For locally advanced EC the guidelines recommend pre‐ and perioperative chemotherapy and/or chemo‐radiotherapy.^9^ For metastatic EC current treatment recommendations are similar to GC, featuring combinations of chemotherapy and anti‐PD1 immunotherapy.^9^, ^10^ For both of these cancers, centralization of treatment, increasing consultation by multidisciplinary teams and optimization of palliative care have been recognized. These were the central part of the DK national cancer program of year 2000 that was soon adopted in NO and SE (but not yet in Finland).^27^

NORDCAN contains no stage or other clinical data but stage‐specific survival data were available from DK and NO covering years 2012–2014 and including GC and EC.^28^ Patients with localized cancer (Stage I and II, 20% of all patients) survived well up to three years, while hardy any patients with Stage IV cancer survived. The Swedish quality register reported survival by clinical stage for GC and EC patients (*N* = 2240 and 3330) diagnosed in 2019–23 (https://statistik.incanet.se/Esofagus‐Ventrikel/). For GC Stage I patients (12.2% of all) 1‐year survival was 71.9%; for Stage II and III patients (22.3 and 15.3% of all) survival was 60.7% and 57.1%, respectively; for Stage IV patients (50.2% of all) survival was 22.6%. Stage‐specific 1‐year survival for EC was 78.2%, 57.8%, 57.6% and 29.3% for Stages I to IV, with respective proportions of cases, 2.3, 11.9, 29.9 and 55.8%.

International data on EC and GC survival for years 2010–2014 have not been particularly favorable for the Nordic countries.^1^ 5‐year survival for EC was between 12 and 17%, and for GC it was between 20 and 32%; US survival was at 20% for EC and 33% for GC. The leading countries for EC were Japan (36%), China (34%) and Korea (31%) and for GC they were Korea (69%) and Japan (60%). DK and NO were part of a more recent study in developed countries on GC and EC with an intermediate survival ranking.^28^ Our most recent US SEER data (2015) show 5‐year survival for EC at 22% for men and 23% for women; GC survival was at 30% and 40%, respectively. These can be compared with the best Nordic survival which for EC was for NO men (23.9%) and women (30.8%) and for GC for NO men (32.3%) and DK women (37.1%). The excellent survival data for East Asia is probably due to endoscopic screening for GC and for EC in China.^29^ In China EC survival for both squamous cell carcinoma and adenocarcinoma have been better in screened compared to non‐screened areas.^30^

Age‐specific survival data have not been included in many survival studies, and in those considering age, the age‐groups have often been wide without specifying the very old (>80 years) patients. However the first study on GC and EC from NORDCAN, covering years 1964 to 2003, included ages up to 90+ years, with 0% survival in most periods.^4^ GC was included in a European study from years 1999 to 2007 showing 5‐year survival among the patients aged over 74 years to be about a half of that for the younger patients.^31^ A recent Dutch study reported that 5‐year survival in EC had not increased in patients older than 79 years between 1990 and 2019 in contrast to more than doubling of survival for younger patients.^32^ A SEER‐based study from years 2012 to 2016 compared age‐group‐specific 1‐year survival and reported 34.8% for GC and 27.4% for EC among patients older than 84 years.^12^

The main limitation in the present data set is the lacking of detailed pathological, clinical and treatment data, which would help a direct interpretation of the age‐group comparisons. As compensation, we have collected relevant clinical data from other sources. We have no direct answers to the observed country‐specific differences which revealed the weakest development in FI, particularly in EC and in male cancers. Table S1 shows that FI economic resources and investment in health care have been below the other countries. Also we noted above that FI has not yet instituted a national cancer program which may signal contentment with the status quo. A further weakness is that the cancer types studied are not common which implies that when patients are divided in age‐groups by periods, sample sizes are small. The upsides of the NORDCAN data are the most recent, high‐quality nationwide datasets spanning a half century.

In conclusion, we could confirm the improving survival for GC and EC for which 1‐year survival was the main driver. Also 5 year survival increased, particularly for EC, suggesting that not only did patients live longer with disease, but more were cured. As a novel observation we could document that the survival improvement were unevenly distributed disfavoring patients older than 79 year. Interestingly, this was in stark contrast to 70–79 year olds, whose survival improved in all countries. Particularly in EC the difference between 70–79 and 80–89 year old was notable, suggesting perhaps that patients over 80 are not diagnosed and treated as actively as patients under 80. Older patients are underrepresented in clinical trials, which may influence what is considered evidence‐based in clinical practice.^14^, ^33^ In EC the survival disparity increased over calendar time and it was wider in 5‐year compared to 1‐year survival. This was in contrast to GC for which the age disparity arose in 1‐year survival and remained almost constant over time. Population campaigns about the early symptoms may alert some at‐risk individuals to seek medical contacts. As treatment of EC and GC continues to be challenging, prevention is of outmost important. In this regard, treatment of *H. pylori* infections and Barrett's esophagus target disease pathways.^6^ Novel therapies, such as improved immunotherapies, and biomarkers for risk and early disease could help reduce mortality in the future.

## AUTHOR CONTRIBUTIONS

**Kari Hemminki:** Conceptualization (lead); formal analysis (equal); investigation (lead); supervision (lead); writing – original draft (lead). **Frantisek Zitricky:** Formal analysis (lead); validation (equal); writing – original draft (supporting). **Asta Försti:** Formal analysis (supporting); validation (equal); writing – original draft (supporting). **Otto Hemminki:** Formal analysis (supporting); validation (supporting); writing – review and editing (equal). **Vaclav Liska:** Conceptualization (supporting); validation (supporting); writing – review and editing (supporting). **Akseli Hemminki:** Conceptualization (supporting); validation (supporting); writing – original draft (supporting); writing – review and editing (supporting).

## FUNDING INFORMATION

Supported by the European Union's Horizon 2020 research and innovation programme, grant No 856620, the Cooperatio Program, research area SURG and National Institute for Cancer Research–NICR (Programme EXCELES, ID Project No. LX22NPO5102), funded by the European Union–Next Generation EU, Jane and Aatos Erkko Foundation, Sigrid Juselius Foundation, Finnish Cancer Organizations, University of Helsinki, Helsinki University Central Hospital, Novo Nordisk Foundation, Päivikki and Sakari Sohlberg Foundation.

## CONFLICT OF INTEREST STATEMENT

A.H. is shareholder in Circio holdings ASA. A.H. is employee and shareholder in TILT Biotherapeutics Oy. Other authors declared no conflict of interest.

## ETHICS STATEMENT

Publically available data without individual identifiers pose no ethical issues.

## Supporting information


Table S1.

## Data Availability

Publically available data at https://nordcan.iarc.fr/en/database#bloc2.
