# Supplementary material for: Survival improvements in esophageal and gastric cancers in the Nordic countries favor younger patients
Source: Cancer Med. 2024 Aug 3;13(15):e7365. doi: 10.1002/cam4.7365 (PMC11297530; doi:10.1002/cam4.7365)
Supplement: Supplementary file 1 — Table S1. [file CAM4-13-e7365-s001.docx]

|  |  |  |  |  |  |  |  |  |  |  |  |
| --- | --- | --- | --- | --- | --- | --- | --- | --- | --- | --- | --- |
| **Supplementary Table 1.** Total health care costs in the Nordic countries between 1970 and 2020 measured as percent of the per person gross national product (GNP, US dollars). | | | | | | | | | | | |
|  |  |  |  |  |  |  |  |  |  |  |  |
| Year | Denmark | |  | Finland | |  | Norway | |  | Sweden | |
|  | US$ | % |  | US$ | % |  | US$ | % |  | US$ | % |
| 1970* | 338 | 7.7 |  | 152 | 5.0 |  | 154 | 4.0 |  | 272 | 5.4 |
| 1980 | 797 | 8.4 |  | 493 | 5.9 |  | 608 | 5.4 |  | 898 | 7.7 |
| 1990 | 1442 | 8.0 |  | 1262 | 7.3 |  | 1361 | 7.1 |  | 1490 | 7.2 |
| 2000 | 2346 | 8.1 |  | 1877 | 7.1 |  | 2794 | 7.7 |  | 2196 | 7.3 |
| 2010 | 4266 | 10.6 |  | 3427 | 9.1 |  | 4777 | 8.9 |  | 3431 | 8.3 |
| 2020 | 5697 | 10.6 |  | 4615 | 9.6 |  | 6582 | 11.2 |  | 5734 | 11.3 |
|  |  |  |  |  |  |  |  |  |  |  |  |
| ^*^The first in Denmark was 1971. | | | | | | | |  |  |  |  |
| Source OECD (2023). Health spending (indicator). doi: 10.1787/8643de7e-en. | | | | | | | | | | |  |

|  | Esophageal cancer | | Gastric cancer | |
| --- | --- | --- | --- | --- |
|  | Males | Females | Males | Females |
| Age group | Denmark | | | |
| 0-49 | 30.3 [19.7 - 46.4] | - | 29.8 [22.5 - 39.4] | 39.2 [29.7 - 51.9] |
| 50-59 | 21.7 [17.3 - 27.1] | 27.6 [18.9 - 40.3] | **30.3 [25.3 - 36.3]** | **46.4 [38.3 - 56.1]** |
| 60-69 | 20.7 [17.8 - 24.1] | 25.3 [20.0 - 31.9] | 33.5 [29.8 - 37.8] | 40.6 [34.8 - 47.5] |
| 70-79 | 20.0 [16.9 - 23.8] | 14.9 [10.9 - 20.3] | 29.5 [25.7 - 33.7] | 36.9 [31.6 - 43.1] |
| 80-89 | 9.0 [5.2 - 15.5] | 6.2 [2.4 - 16.1] | 21.4 [15.8 - 28.9] | 18.8 [12.4 - 28.6] |
|  | Finland | | | |
| 0-49 | 15.5 [8.0 - 29.9] | - | 39.8 [30.5 - 52.1] | 54.0 [44.0 - 66.3] |
| 50-59 | 17.8 [12.6 - 25.2] | 15.9 [8.6 - 29.5] | 31.4 [25.3 - 39.0] | 44.5 [36.8 - 53.9] |
| 60-69 | 17.9 [14.6 - 21.9] | 22.3 [16.0 - 31.0] | 26.6 [22.8 - 31.0] | 34.7 [29.5 - 41.0] |
| 70-79 | **13.8 [10.4 - 18.2]** | **29.3 [22.3 - 38.4]** | 28.4 [24.3 - 33.3] | 33.9 [28.7 - 39.9] |
| 80-89 | 4.8 [1.1 – 21.5] | 5.0 [2.1 - 11.8] | 20.9 [15.3 - 28.5] | 17.3 [12.6 - 23.8] |
|  | Norway | | | |
| 0-49 | 40.5 [29.6 - 55.4] | - | 44.6 [34.8 - 57.2] | 46.5 [36.2 - 59.5] |
| 50-59 | 25.5 [19.1 - 34.0] | 30.8 [18.5 - 51.3] | 36.9 [29.7 - 45.9] | 34.7 [26.5 - 45.5] |
| 60-69 | 26.2 [21.8 - 31.5] | 35.7 [26.7 - 47.6] | 30.9 [26.2 - 36.4] | 37.4 [30.4 - 46.0] |
| 70-79 | 22.5 [18.1 - 27.9] | 28.6 [21.1 - 38.6] | 30.7 [26.2 - 36.0] | 30.4 [24.4 - 37.9] |
| 80-89 | 7.2 [3.5 - 14.8] | 15.8 [8.0 - 31.2] | 23.0 [16.7 - 31.5] | 18.2 [12.1 - 27.5] |
|  | Sweden | | | |
| 0-49 | 32.1 [20.9 - 49.1] | - | 36.6 [29.5 - 45.4] | 45.4 [37.6 - 54.8] |
| 50-59 | 20.0 [14.3 - 28.0] | 29.0 [19.6 - 43.0] | 34.5 [29.3 - 40.7] | 37.9 [30.6 - 46.8] |
| 60-69 | 20.1 [16.7 - 24.2] | 24.8 [19.0 - 32.5] | 32.0 [28.3 - 36.2] | 34.2 [29.1 - 40.1] |
| 70-79 | 16.1 [13.2 - 19.7] | 15.9 [11.6 - 21.9] | 26.9 [23.8 - 30.4] | 33.1 [28.7 - 38.2] |
| 80-89 | 8.1 [4.8 - 14.0] | 11.1 [5.7 – 21.3] | 17.8 [13.6 - 23.3] | 19.6 [15.0 - 25.7] |

**Supplementary Table 2.** 5-year age specific relative survival [95 % CI] in esophageal and gastric cancers by sex (2017-2021). Bold marks significant difference in relative survival between male and female cohort.
